# Molecular Dynamics, Monte Carlo Simulations, and Langevin Dynamics: A Computational Review

**DOI:** 10.1155/2015/183918

**Published:** 2015-02-16

**Authors:** Eric Paquet, Herna L. Viktor

**Affiliations:** ^1^Vaccine Program, National Research Council, 1200 Montreal Road, Ottawa, ON, Canada K1A 0R6; ^2^School of Electrical Engineering and Computer Science, University of Ottawa, 800 King Edward Road, Ottawa, ON, Canada K1N 6N5

## Abstract

Macromolecular structures, such as neuraminidases, hemagglutinins, and monoclonal antibodies, are not rigid entities. Rather, they are characterised by their flexibility, which is the result of the interaction and collective motion of their constituent atoms. This conformational diversity has a significant impact on their physicochemical and biological properties. Among these are their structural stability, the transport of ions through the M2 channel, drug resistance, macromolecular docking, binding energy, and rational epitope design. To assess these properties and to calculate the associated thermodynamical observables, the conformational space must be efficiently sampled and the dynamic of the constituent atoms must be simulated. This paper presents algorithms and techniques that address the abovementioned issues. To this end, a computational review of molecular dynamics, Monte Carlo simulations, Langevin dynamics, and free energy calculation is presented. The exposition is made from first principles to promote a better understanding of the potentialities, limitations, applications, and interrelations of these computational methods.

## 1. Introduction

**Figure 1:**
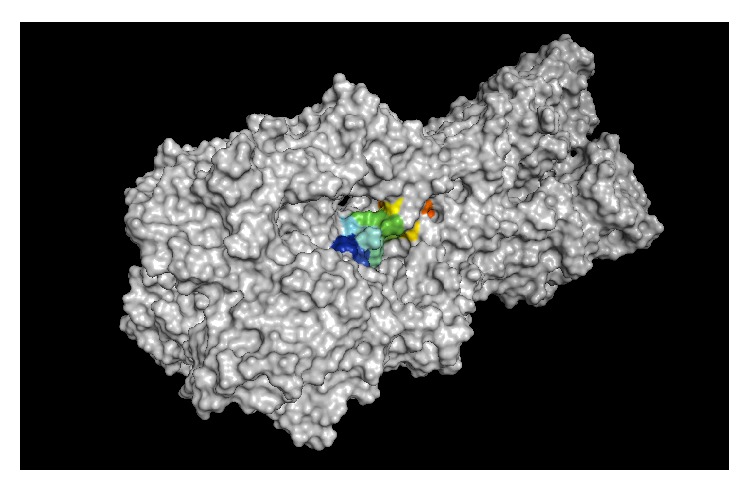
Accessibility assessment of a region of the influenza A virus (A/swine/Iowa/15/1930 (H1N1)).

The ability to properly sample configurational and conformational properties and to subsequently describe at the atomic level the dynamical evolution of complex macromolecular systems has wide application. This research is of paramount importance in the study of macromolecular stability of mutant proteins [1], molecular recognition, ions, and small molecule transportation of the influenza M2 channel [2, 3], protein association, the role of protein flexibility for influenza A RNA binding [4, 5], folding and hydration, influenza neuraminidase inhibitor [6–9], drug resistance [10], enzymatic reactions, folding transitions [11, 12], screening [13], accessibility assessment (see Figure 1), and hemagglutinin fusion peptide [14]. One should also mention multivalent binding mode [15], docking [16], drug (e.g., Oseltamivir and Zanamivir) efficiency against mutants [17, 18], structural biochemistry [19], biophysics, molecular biology, influenza multiple dynamics interactions [20], enzymology, pharmaceutical chemistry [21], biotechnology, rational epitope design [22], computation vaccinology [23], binding [24], and free energy [25, 26]. For instance, one may wish to calculate the free energy to assess the strength and the stability of the bond in between a monoclonal antibody (mAb) and an antigen, such as the viral hemagglutinin, to quantify the efficiency of the neutralisation process.

This paper presents an algorithmic review from the first principles of Monte Carlo simulation, molecular dynamics, and Langevin dynamics (i.e., techniques that have been shown to address the abovementioned scenario). We focus our attention on the algorithmic aspect, which, within the context of a review, has not received sufficient attention. Our objective is not only to explain the algorithms but also to highlight their potential, limitations, applicability, interrelations, and generalisation in the context of molecular dynamics. To this end, a number of algorithmic approaches are presented in detail, and the pros and cons of each are highlighted. The algorithms are illustrated with examples related to the influenza virus.

This paper is organised as follows. Monte Carlo simulations are reviewed in Section 2.  Section 3 is concerned with molecular dynamics in the microcanonical ensemble, that is, at constant energy.  Section 4 extends molecular dynamics to the canonical and the isobaric-isothermal ensemble. Constrained molecular dynamics, hybrid molecular dynamics, and steered molecular dynamics are also presented.  Section 5 introduces Langevin and self-guided Langevin dynamics, and Section 6 is concerned with the calculation of the free energy. The application of molecular dynamics to macromolecular docking is addressed in Section 7. Finally, the connection in between molecular dynamics and quantum mechanics (ab initio simulations) is outlined in Section 8. This is followed by a short conclusion.

## 2. Monte Carlo Simulations

The objective of a Monte Carlo (MC) simulation is to generate an ensemble of representative configurations under specific thermodynamics conditions for a complex macromolecular system [27]. Applying random perturbations to the system generates these configurations. To properly sample the representative space, the perturbations must be sufficiently large, energetically feasible and highly probable. Monte Carlo simulations do not provide information about time evolution. Rather, they provide an ensemble of representative configurations, and, consequently, conformations from which probabilities and relevant thermodynamic observables, such as the free energy, may be calculated. Monte Carlo simulations are not only important on their own right, but they also play a fundamental role when designing complex and hybrid molecular dynamic (MD) algorithms [28].

This section is dedicated to Monte Carlo simulations. In Section 2.1 we review some important notions about Lagrangian and Hamiltonian dynamics, which are pervasive for both Monte Carlo simulations and molecular dynamics. In Section 2.2 we introduce the partition function and the probability density function, as well as the calculation of thermodynamics observable associated with a macromolecule such as the hemagglutinin or the neuraminidase. The partition function is instrumental in computing such observables. In Section 2.3 we explain how to efficiently sample the representative space. For that, we introduce the notions of emission probability, transition probability, acceptance probability, and detailed balance.

Sampling is useful only when performed in realistic experimental conditions. For this reason we explain how to sample in the canonical ensemble (with a constant number of particles, volume, and temperature) and also in the isothermal-isobaric ensemble (with a constant number of particles, pressure, and volume) in Sections 2.4 and 2.5, respectively. Finally, in Section 2.6 we address the problem of sampling in the presence of numerous minima. This is a problem particularly acute when studying influenza macromolecular structures such as the hemagglutinin and the neuraminidase.

### 2.1. Lagrangian and Hamiltonian Dynamics or How to Formulate Our Problem

This section presents some important notions about Lagrangian and Hamiltonian dynamics, which are pervasive and recurrent for both MC and MD. Lagrangian and Hamiltonian dynamics provide an ideal framework for the description of complex macromolecular systems, both in Cartesian and generalised coordinates [29]. The Lagrangian is defined as the difference in between the kinetic and the potential energy:

(1) $$\begin{array}{c} L\left(q^{3N},{\dot{q}}^{3N}\right)=K\left({\dot{q}}^{3N}\right)-U\left(q^{3N}\right), \end{array}$$

where the kinetic energy is given by

(2) $$\begin{array}{c} K=\overset{3N}{\underset{\alpha =1}{\sum }}\frac{1}{2}m_{\alpha }{\dot{q}}_{\alpha }^{2}, \end{array}$$

and potential energy *U*(*q* ^3*N*^) is a function of the positions of the constituent atoms. The *q* ^3*N*^ are the generalised coordinates, and the ${\dot{q}}^{3N}$ are the generalised velocities. For instance, a generalised coordinate may be a bound length, a bound angle, or a dihedral angle. The space of all generalised coordinates and velocities is called the configuration space. If one introduces the generalised momentum:

(3) $$\begin{array}{c} p^{\alpha }\equiv \frac{\partial L}{\partial {\dot{q}}_{\alpha }}, \end{array}$$

one may define the Hamiltonian, which is the Legendre transformation of the Lagrangian:

(4) $$\begin{array}{c} H\left(q^{3N},p^{3N}\right)=\overset{3N}{\underset{\alpha =1}{\sum }}p^{\alpha }q_{\alpha }-L\left(q^{3N},{\dot{q}}^{3N}\left(q^{3N},p^{3N}\right)\right). \end{array}$$

The Hamiltonian obeys the so-called Hamilton's equations, which is just another formulation of Newton's equations:

(5) $$\begin{array}{c} \frac{dH}{dt}=0;\;\;{\dot{q}}_{\alpha }=\frac{\partial H}{\partial p^{\alpha }};\;\;{\dot{p}}^{\alpha }=-\frac{\partial H}{\partial q_{\alpha }}. \end{array}$$

Throughout the text, we will use both generalised and Cartesian coordinates: the Cartesian coordinates of the *i* constituent atom are noted **r**_*i*_ ≡ [*x*_*i*_, *y*_*i*_, *z*_*i*_]^*T*^. The set of all Cartesian coordinates is noted as

(6) $$\begin{array}{c} r^{N}\equiv r_{1},\ldots ,r_{N}, \end{array}$$

and the associated differential volume element is expressed as

(7) $$\begin{array}{c} dr^{N}\equiv dr_{1}\ldots dr_{N}. \end{array}$$

In the next section, we introduce the notions of partition function and probability density function.

### 2.2. Partition Functions, Probability Density Functions, and Expectation or How to Compute Observables

Partition functions are pervasive to all Monte Carlo simulations. They are required to determine the number of microstates associated with a macromolecule, the probability of occurrence of a specific conformation, and the ensemble averages of observables, such as the enthalpy or thermodynamical quantities like the free energy from which the strength of a bound between a drug, such as Oseltamivir [30], and a viral neuraminidase may be asserted.

The number of microstates may be obtained by

(8) $$\begin{array}{c} \Omega \left(N,V,E\right)\equiv E_{0}C_{\left\{N\right\}}\int dp^{N}dr^{N}\delta \left(H\left(r^{N},p^{N}\right)-E\right), \end{array}$$

where

(9) $$\begin{array}{c} C_{\left\{N\right\}}=\frac{1}{h^{3N}\left(N_{A}!N_{B}!\cdots \right)} \end{array}$$

is a quantum factor, which accounts for the indiscernibility of the various atomic species *A*,  *B*, and so forth, *h* is the Planck constant, and *δ*(*x*) is Dirac delta function. The function *Ω*(*N*, *V*, *E*) counts the number of states of constant energy *E* in a system. It is directly related to the entropy

(10) $$\begin{array}{c} S\left(N,V,E\right)=k_{B}\ln\Omega \left(N,V,E\right), \end{array}$$

where *k*_*B*_ is the Boltzmann constant. The canonical partition function is defined as

(11) $$\begin{array}{c} Z\left(N,V,T\right)\equiv \int dp^{N}dr^{N}\exp\left[-\beta H\left(r^{N},p^{N}\right)\right], \end{array}$$

where

(12) $$\begin{array}{c} \beta \equiv \frac{1}{k_{B}T} \end{array}$$

and *T* is the temperature. The canonical partition function is a functional, which is uniquely determined by the Hamiltonian of the corresponding macromolecular system. If the indiscernibility factor is included in the definition, the microcanonical partition function is noted as

(13) $$\begin{array}{c} Q\left(N,V,T\right)\equiv C_{\left\{N\right\}}Z\left(N,V,T\right). \end{array}$$

The probability that the macromolecule is in a state characterised by atomic positions **r** ^*N*^, and atomic momenta **p** ^*N*^ is given by

(14) $$\begin{array}{c} \mathrm{Pr}\left(r^{N},p^{N}\right)dp^{N}dr^{N}=\frac{\exp\left[-\beta H\left(r^{N},p^{N}\right)\right]dp^{N}dr^{N}}{Z\left(N,V,E\right)}. \end{array}$$

Consequently, the ensemble average of an observable *O* (such as the enthalpy) is obtained by weighting the various realisations of the observable by their corresponding probability:

(15) $$\begin{array}{c} \langle O\rangle =\int dp^{N}dr^{N}O\left(r^{N},p^{N}\right)\mathrm{Pr}\left(r^{N},p^{N}\right). \end{array}$$

The uncertainty (standard deviation) associated with the observable is given by

(16) $$\begin{array}{c} \sigma \left(O\right)=\sqrt{\langle O^{2}\rangle -{\langle O\rangle }^{2}}. \end{array}$$

Unfortunately, it is not possible to integrate the partition function or to compute the probability directly. This is due to the large number of degrees of freedom. For instance, the* Homo sapiens* influenza hemagglutinin is formed of approximately 23.000 atoms, which means that the partition function must be integrated in a 138.000-dimensional space.

In the next section, we explain how to perform such integration efficiently.

### 2.3. Stochastic Sampling or How to Sample Efficiently Thermodynamical Quantities

The multidimensional integrals associated with the probability and the partition function may be efficiently calculated with a procedure called Monte Carlo integration. In this approach the integration space is sampled according to a Markovian process and the integral is approximated by the average of the corresponding sampled states. Such an approach is efficient if the sampled states have a high probability of occurrence.

A sufficient, but not necessary, condition for such an efficient sampling to hold is called detailed balance:

(17) Pr⁡ITI⟶JAI⟶J =Pr⁡JTJ⟶IAJ⟶I,

where *Pr*⁡(*I*) is the probability (emission probability) that the system is in the state *I* ≡ {**r** ^*N*^, **p** ^*N*^}, *T*(*I* → *J*) is the transition probability from state *I* to state *J*, and *A*(*I* → *J*) is the acceptance probability of such a transition. If we assume that the transition probability is symmetrical

(18) $$\begin{array}{c} T\left(I⟶J\right)=T\left(J⟶I\right), \end{array}$$

then the detailed balance equation reduces to

(19) $$\begin{array}{c} \frac{A\left(I⟶J\right)}{A\left(J⟶I\right)}=\frac{\mathrm{Pr}\left(J\right)}{\mathrm{Pr}\left(I\right)}=\exp\left[-\beta \left(U\left(J\right)-U\left(I\right)\right)\right]. \end{array}$$

A possible solution for this equation is

(20) $$\begin{array}{c} A\left(I⟶J\right)=\min\left\{1,\exp\left[-\beta \left(U\left(J\right)-U\left(I\right)\right)\right]\right\}. \end{array}$$

This equation is the celebrated Metropolis algorithm [31]. Consequently, each state is defined from the previous one (Markovian process). A transition to a lower energy is always accepted, while a transition to a higher energy is accepted with probability exp⁡⁡[−*β*(*U*(*J*) − *U*(*I*))].

Numerous variations have been designed based on this algorithm [32, 33]. For instance, the local elevation method enhances sampling by adding penalty potential to any state previously sampled (also known as a taboo search algorithm). Although useful, the microcanonical partition function is not realistic from an experimental point of view. Indeed, most observations are performed either at constant volume and temperature (canonical ensemble) or at constant pressure and temperature (isobaric-isothermal ensemble). These distributions are introduced in the next two sections.

### 2.4. Canonical Ensemble (NVT) Sampling or How to Sample in Realistic Experimental Conditions

The canonical ensemble is the ensemble associated with the observations made at constant volume and constant temperature. The configurational canonical partition function associated with such an ensemble is obtained by marginalising the momenta in (11):

(21) $$\begin{array}{c} Z\left(N,V,T\right)\equiv \int dr^{N}\exp\left[-\beta U\left(r^{N}\right)\right]. \end{array}$$

It may also be defined as

(22) $$\begin{array}{c} Q\left(N,V,T\right)\equiv M_{\left\{N\right\}}Z\left(N,V,T\right), \end{array}$$

where the constant

(23) $$\begin{array}{c} M_{\left\{N\right\}}=\frac{1}{{\left(\sqrt{h^{2}\beta /2\pi m_{A}}\right)}^{3N_{A}}N_{A}!{\left(\sqrt{h^{2}\beta /2\pi m_{B}}\right)}^{3N_{B}}N_{B}!\cdots } \end{array}$$

takes into account the indiscernibility of the constituent atoms. Like in the microcanonical ensemble, the probability of occurrence of a given state {**r** ^*N*^} is equal to

(24) $$\begin{array}{c} \text{P}{\text{r}}_{NVT}\left(r^{N}\right)dr^{N}=\frac{\exp\left[-\beta U\left(r^{N}\right)\right]dr^{N}}{Z\left(N,V,T\right)}. \end{array}$$

Consequently, the average value of an observable is given by

(25) $$\begin{array}{c} \langle O\rangle =\frac{1}{Z\left(N,V,T\right)}\int dr^{N}\exp\left[-\beta U\left(r^{N}\right)\right]O\left(r^{N}\right). \end{array}$$

In the case of the canonical partition function, the acceptance probability associated with Monte Carlo method reduces to

(26) ANVTrN⟶r′N  =min⁡1,exp⁡−βUr′N−UrN.

From the canonical partition function, it is possible to obtain various thermodynamical quantities such as the Helmholtz free energy:

(27) $$\begin{array}{c} F\left(N,V,T\right)=-k_{B}T\ln Q\left(N,V,T\right). \end{array}$$

Still, most observations are performed at constant pressure and temperature. To address this limitation, the isobaric-isothermal ensemble, as presented in the next section, is introduced.

### 2.5. Isobaric-Isothermal Ensemble (NPT) Sampling or How to Sample in Even More Realistic Experimental Conditions

The isobaric-isothermal ensemble is representative of many experimental conditions. From the microcanonical formalism, it is possible to demonstrate that the isobaric-isothermal configurational partition function is equal to

(28) $$\begin{array}{c} Z\left(N,P,T\right)\equiv \int dV\exp\left[-\beta PV\right]\int dr^{N}\exp\left[-\beta U\left(r^{N}\right)\right]. \end{array}$$

As usual, if the indiscernibility of the constituent atoms is taken into account, the partition function becomes

(29) $$\begin{array}{c} Q\left(N,P,T\right)=\frac{M_{\left\{N\right\}}}{V_{0}}Z\left(N,P,T\right). \end{array}$$

Such a partition function assumes that the deformations of the macromolecular structure are isotropic (the same in all directions). These deformations occur to maintain the pressure constant. If the deformations are anisotropic, the partition function must be modified as follows:

(30) $$\begin{array}{c} Z\left(N,P,T\right)=\int dHZ^{\prime }\left(N,P,T,H\right)\delta \left(\det H-V\right), \end{array}$$

where **H** is the tensor associated with an elementary parallelepiped volume, which must be integrated (marginalised) over all possible variations of the elementary shape. The probability that a macromolecular system is in a state **r** ^*N*^ is given by

(31) $$\begin{array}{c} \text{P}{\text{r}}_{NPT}\left(r^{N}\right)dr^{N}=\frac{\exp\left[-\beta PV\right]\exp\left[-\beta U\left(r^{N}\right)\right]dr^{N}}{Z\left(N,P,T\right)}. \end{array}$$

Again, various thermodynamical quantities may be defined from the partition function such as the Gibbs free energy:

(32) $$\begin{array}{c} G\left(N,P,T\right)=-k_{B}T\ln Q\left(N,P,T\right). \end{array}$$

The isobaric-isothermal acceptance probability associated with the Monte Carlo method is

(33) ANPTrN,V⟶r′N,V′ =min⁡−βPV′−V+Nln⁡V′V1,exp⁡−βUr′N,V′−UrN,Vmmmmmm×exp⁡−βPV′−V+Nln⁡V′V.

Irrelevant of the ensemble in which the calculations are performed, the Metropolis algorithm may be impaired by local minima [27]. Indeed, the acceptance probability may become trapped in a local minimum of the potential energy, which may result in an inadequate sampling of the macromolecular states, as seen in the conformational states [32, 33]. This issue is addressed in the following section.

### 2.6. Sampling and Local Minima or When Temperature May Help to Escape Local Minima

Many biomolecular processes associated with influenza involve activated processes in which a high-energy barrier exists in between the initial and the final state [34]. In order to efficiently sample the macromolecular states, this type of barrier must be overcome.

An efficient, although computationally expensive, approach to overcome such a barrier is called replica exchange (refer to [34] and, in the same spirit, [35]). These methods involve a certain number of noninteracting simulations, called replicas, which are performed in parallel. Each simulation is characterised by its own temperature: low temperature simulations tend to explore local minima, while high temperature simulations may overcome energy barriers and consequently move in between local minima. To favour a better exploration of the macromolecular states, the replicas are periodically exchanged (swapped) according to the following acceptance probability:

(34) ARI⟶J =min⁡I,exp⁡−βJ−βImmmmnimmmm×UJTJ−UITI,

where

(35) $$\begin{array}{c} \beta_{I}\equiv \frac{1}{k_{B}T_{I}}. \end{array}$$

This acceptance probability is similar to the ones introduced before, except for the fact that each state is characterised by its own temperature. Once the exchange is completed, the simulations resume normally until another exchange is performed. The whole procedure allows for a better sampling of the macromolecular states. For instance, this approach has been utilised recently, in conjunction with simulated annealing, for creating the infectious disease model of the H1N1 influenza pandemic [36].

Until now, we have restricted ourselves to symmetrical transition functions. Often, a better sampling may be obtained if a nonsymmetrical sampling function is employed. Let us consider the particular case in which the nonsymmetrical function depends uniquely on the final conformation:

(36) $$\begin{array}{c} T\left(I⟶J\right)=\pi \left(U\left(J\right)\right). \end{array}$$

Then, the acceptance probability becomes

(37) AI⟶J =min⁡1,πUIπUJexp⁡−βUJ−UI.

This is the so-called bias sampling algorithm [37], which considerably increases the conformational sampling efficiency of large macromolecular chains [38].

Although MC simulations allow us to sample the most probable macromolecular states, they do not provide us with their temporal evolution. The study of the temporal evolution of a macromolecular state is called molecular dynamics and is the subject of the next section.

## 3. Molecular Dynamics or When Time Matters

Molecular dynamics studies the temporal evolution of the coordinates and the momenta (the state) of a given macromolecular structure. Such an evolution is called a trajectory. A typical trajectory is obtained by solving Newton's equations. The trajectory is important in assessing numerous time-dependent observables [39] such as the accessibility of a given molecular surface [40], the interaction in between a small molecule (e.g., a drug) and the hemagglutinin or the neuraminidase of a given influenza strain, the interaction epitope-paratope in between an antigen (e.g., hemagglutinin) and an antibody (e.g., CR8020), the appearance and disappearance of a particular channel or cavity, and the fusion of the hemagglutinin with a cell membrane (fusion peptide), amongst others.

From an MD trajectory, it is possible to compute a temporal average of an observable by averaging this observable over time along the trajectory:

(38) $$\begin{array}{c} \overset{-}{O}=\underset{t\to \infty }{\lim}\frac{1}{t}\overset{t}{\underset{0}{\int }}d\tau O\left(r\left(\tau \right),\dot{r}\left(\tau \right)\right). \end{array}$$

Although it has never been formally proven (and that it is not always applicable: for instance, when the trajectory is periodic or when the phase space is constituted of disconnected regions), the ergodicity principle is often invoked [41]. The ergodicity principle states that the average over periods of time along a given trajectory of an observable is, at the limit, identical to the ensemble average of this observable as obtained, for instance, from Monte Carlo simulations:

(39) $$\begin{array}{c} \overset{-}{O}\approx \langle O\rangle . \end{array}$$

As we will see later, ergodicity is instrumental in performing MD simulations in the canonical and isobaric-isothermal ensemble. The next section is devoted to the potential or force field.

### 3.1. Potential or How to Approximate the Force Field

The choice of a proper potential is of the utmost importance in obtaining accurate molecular dynamics simulations [42]. The potential must be physically sound as well as computationally tractable. An approximate potential may be calculated from quantum mechanics and from the Born-Oppenheimer approximation in which only the positions of the atomic nucleus bonding are considered [42]. The potentials may be divided into bonding potentials and long-range potentials. The bonding potentials involve interaction with two atoms (bound lengths), three atoms (bound angles), and four atoms (dihedral angles). Long-range interactions are associated with the Lennard-Jones potential (van der Waal) and the Columbic potential. The harmonic approximation is utilised for the bonding potentials, which means that solely small displacements are accurately represented. The general form of the potential is

(40) UrN=∑dkdd−d02+∑SkSS−S02+∑θkθθ−θ02+∑χkχ1+cos⁡nχ−δ+∑φkφφ−φ02+∑i,jεijrij0rij12−rij0rij6+qiqjεlrij,

where *d* is the bound length, *S* is the Urey-Bradley bound length, *θ* is the bound angle, *χ* is the dihedral angle, *φ* is the improper dihedral angle, *r*_*ij*_ is the distance in between atom *i* and *j*, *k*_*d*_, *k*_*S*_, *k*_*θ*_, *k*_*χ*_, and *k*_*φ*_ are constants, *d*_0_, *S*_0_, *θ*_0_, *φ*_0_, and *r*_*ij*_^0^ are equilibrium positions, *ε*_*ij*_ is related to the Lennard-Jones well depth, and *ε*_*l*_ is the effective dielectric constant. Finally, *q*_*i*_ is the partial atomic charge associated with atom *i*: the partial charge comes from the asymmetrical distribution of the electrons in the chemical bounds. The first term on the last line is the van der Waal interaction (or Lennard-Jones potential), and the last term on the last line is the Columbic interaction. The parameters of the model are determined experimentally and from quantum mechanics. Among the most popular potentials are CHARMM and AMBER [42, 43]. The two differ mostly in the manner in which the parameters are estimated. These potentials may model proteins, lipids, ethers, and carbohydrates, as well as small molecules (e.g., drugs).

The number of interactions involved in long-range interactions rapidly becomes prohibitive. For instance, for the Columbic potential, there are potentially *N*!/2!(*N* − 2)! interactions, which correspond to approximately a quarter of billon interactions for an influenza hemagglutinin. To reduce the computational burden, their action range is truncated. The truncation should be performed in such a way as not to introduce artificial discontinuities, which may result in computational artefacts.

The next section is concerned with the refinement of experimentally determined macrostructures.

### 3.2. How to Minimize the Energy of the Conformation or How to Refine Experimentally Determined Structures

The position of the constituent atoms of a macromolecular structure is usually determined either through X-ray crystallography for the larger structure or through nuclear magnetic resonance (NMR) for the smaller molecules. If only the amino acid sequence is available, the three-dimensional structure may be inferred either from methods based on homology, such as threading, or from ab initio methods, which predict the structure from the sequence alone [44]. Among the larger structures associated with influenza are the hemagglutinin and the neuraminidase. Because a protein has to be crystallised to apply X-ray crystallography, the position of its constituent atoms may be distorted from their natural positions by the crystallisation process. Consequently, bond lengths and bond angles may be distorted and steric clashes in between atoms may occur. Therefore, it is recommended to minimise the potential energy of the macromolecular structure to remediate this deficiency and to create a more realistic structure [45].

The global optimisation of nonlinear functions, such as the potential, is a notoriously difficult problem because of the complexity of the energy landscape and the profusion of local minima [46]. Usually, only local optimisation is performed. Such a minimisation may be achieved through various algorithms [46] such as the steepest descent algorithm, the conjugate gradient algorithm, and the Newton-Raphson method. The first two are based on the gradient, while the latter is based on the Hessian. In most cases a local optimisation is sufficient to refine the structure.

If a global optimisation is suited or required, an approach such as simulated annealing must be utilised [47]. Simulated annealing is an MC method. The position of the atoms is subjected to small random displacements. The acceptance probability of such a displacement is given by

(41) AI⟶J =min⁡1,exp⁡−βkUJTk−UITk,

where

(42) $$\begin{array}{c} T_{k}\in \left\{T_{1},\ldots ,T_{K}\right\}\;|\;T_{k+1}<T_{k}. \end{array}$$

This means that the temperature acts as a control parameter. Initially, the temperature is high, which implies that transitions from lower to higher energy are allowed with nonnegligible probability in being able to escape local minima. Subsequently, the temperature is gradually reduced (cooling) to decrease the occurrence of such a transition. Transitions to lower energy are always accepted. With a proper choice of temperatures, a global optimisation may be achieved. The position of the global minimum associated with the energy landscape may be further refined with local optimisation.

The next section is devoted to the solvation of macromolecules.

### 3.3. Implicit and Explicit Solvation, Ions, and Poisson-Boltzmann Equation or How to Obtain Realistic Experimental Conditions

Macromolecules do not exist in isolation. Water molecules and ions surround them. Often, to obtain a realistic simulation, the structure of interest must be solvated. Solvation is a vast and complex subject and we refer the reader to the literature for technical details [4, 44, 45, 48]. This section is devoted to some aspects of solvation, which are particularly relevant to MD.

The solvation may be either implicit or explicit. In the case of an implicit solvation [11], the water molecules are replaced by a potential, which describe their average action while, in the case of an explicit solvation, the macromolecule is surrounded by a solvation box constituted of water molecules. It follows that computers have a limited amount of memory, and thus, the size of this box cannot be infinite. To reduce the number of water molecules, various shapes may be utilised such as cubic, rhombic, dodecahedron truncated octahedron, and sphere. The shape of the solvation box is chosen to minimise the number of molecules required for solvation while maintaining at all times a minimum buffer of solvent. Because of its finite dimensions, the solvation box presents unnatural boundary effects, which should be minimised. This may be partially achieved with a larger solvation box or with periodic boundary conditions (PBC). For a rectangular solvation box, periodic boundary conditions are defined as

(43) $$\begin{array}{c} {\left.O\left(x_{i}\right)=O\left(x_{i}+L_{x}\right)\right|}_{i=1,\ldots ,N},\\ {\left.O\left(y_{i}\right)=O\left(y_{i}+L_{y}\right)\right|}_{i=1,\ldots ,N},\\ {\left.O\left(z_{i}\right)=O\left(z_{i}+L_{z}\right)\right|}_{i=1,\ldots ,N}. \end{array}$$

Much care must be taken when using periodic boundary conditions to avoid unphysical artefacts. For instance, if the boundary box is too small, the head of a macromolecule, such as the hemagglutinin, may interact with its own tail, which is extremely unrealistic. Also, if Columbic interactions are involved, the system must be electrostatically neutral; otherwise, the total charge becomes infinite due to the endless replication of the system associated with the PBC. Ions could be added to the solvation box to neutralise the system. Even if the latter is neutral, ions, such as sodium and chloride, may be added to reproduce the ionic strength of the solvent in which the macromolecule evolves. Due to the periodic nature of the boundary conditions, duplicate interactions may appear. It is customary to apply the minimum image convention in which such duplicate interactions are not allowed. If the structure is too large, the solvation may be limited to a specific region of interest: for example, a binding site or a channel while implicit solvation may be utilised for the remaining part of the macromolecule. There are various solvation models [49–52]. Generally, their parameters are adjusted to reproduce the enthalpy of vaporisation and density of water.

The solvation box increases the complexity of the simulation. Indeed, most of the computational effort is directed toward simulating the solvent. Nevertheless, dielectric screening, electrostatic effects, and free energy, among others, may only be simulated through explicit solvation, which, consequently, is amply justified [53] though one should notice that explicit solvation does not allow for the simulation of the solvent viscosity.

If the macromolecule is solvated in an ionic solution, the electrostatic potential *ϕ* may be obtained with a greater accuracy by solving the Poisson-Boltzmann equation:

(44) ∂∂r·εr∂ϕr∂r =−ρr−∑k=1Kqici∞λrexp⁡−βqiϕr,

where *ρ*(**r**) represents the charge density of the solute (the macromolecule),  *q*_*i*_  is the ionic charge,  *c*_*i*_^*∞*^  is the ionic concentration far from the solute, *ε*(**r**) is the dielectric permittivity, and *λ*(**r**) is an accessibility factor. The Poisson-Boltzmann equation is often used in modelling implicit solvation. It may be solved efficiently with finite element methods (FEM) [54].

In the next section, we show how to solve Newton's equation to obtain the trajectory of a macromolecule.

### 3.4. Integration of Newton's Equations: When Technical Details Matter

To obtain the trajectory associated with a given macromolecule, one should solve the corresponding Newton's equations. This is appropriate, since the trajectories are assumed to follow the laws of classical mechanics [55]. In this section we present a completely general approach from which most finite difference algorithms may be derived.

Newton's equation and their generalisation, Hamilton's equations, present two important characteristic: they are time-reversible and any infinitesimal volume in phase space (space of all coordinates and momenta) is conserved with time. The latter property is known as the Liouville theorem [56]:

(45) $$\begin{array}{c} d^{3N}p\left(0\right)d^{3N}q\left(0\right)=d^{3N}p\left(t\right)d^{3N}q\left(t\right). \end{array}$$

This is another formulation of the conservation of the total energy of the system. Any numerical algorithm must enforce these two properties at all times to be physically realistic and consequently relevant. When aiming to derive a finite different algorithm that complies with these requirements, we start with the Liouville operator defined as

(46) $$\begin{array}{c} iL\equiv \overset{3N}{\underset{\alpha =1}{\sum }}\left[\frac{\partial H}{\partial p^{\alpha }}\frac{\partial }{\partial q_{\alpha }}-\frac{\partial H}{\partial q_{\alpha }}\frac{\partial }{\partial p^{\alpha }}\right], \end{array}$$

where $i=\sqrt{-1}$. This operator allows recasting Hamilton's equations in the form:

(47) $$\begin{array}{c} q^{3N}\left(t\right)=\exp\left[iLt\right]q^{3N}\left(0\right), \end{array}$$

which is known as the Liouville equation. In Cartesian coordinates the Liouville operator becomes

(48) $$\begin{array}{c} iL=iL_{1}+iL_{2},\\ iL_{1}\equiv \overset{N}{\underset{i=1}{\sum }}\frac{p_{i}}{m_{i}}\cdot \frac{\partial }{\partial r_{i}};\;\;iL_{2}\equiv \overset{N}{\underset{i=1}{\sum }}\left(-\frac{\partial U\left(r^{N}\right)}{\partial r_{i}}\right)\cdot \frac{\partial }{\partial p_{i}}. \end{array}$$

Because the two parts of the Liouville operator do not commute *L*_1_ *L*_2_ ≠ *L*_2_ *L*_1_, one must rely on the Trotter theorem [5] to develop the argument of the exponential:

(49) exp⁡iLΔt =exp⁡iL1+L2Δt ≈exp⁡iL2Δt2exp⁡iL1Δtexp⁡iL2Δt2  +OΔt3.

Then, if each exponential is approximated in terms of a truncated Taylor expansion, the Liouville equation becomes

(50) rit+Δt =2rit−rit−Δt  −1mi∂UrNt∂riΔt2+OΔt4i=1,…,N,

which is the well-known Verlet algorithm [1]. Note that other common algorithms, such as the Leap Frog algorithm and the reference system propagator algorithm (RESPA), may be derived following a similar approach [6, 7]. The Liouville operator may be partitioned in more than one way. For instance, the potential may be divided into short- and long-range potentials (e.g., Coulombs and van der Waal). Since long-range potentials tend to change more slowly than short-range potentials, a larger time increment may be used for the former. If this procedure is repeated, a multiple time-step algorithm may be designed. Once the trajectory has been calculated, the functional important motions may be separated from the random thermal motion by performing a principal component analysis (PCA), which expresses the dominant modes as linear combinations of the underlying motion [57].

The algorithms described in this section are only valid when the total energy is conserved, that is, in the microcanonical ensemble. In next section we show how to extend this approach to the experimentally more realistic canonical and isobaric-isothermal ensembles.

## 4. Non-Hamiltonian Molecular Dynamics or How to Reproduce Realistic Experimental Conditions during Molecular Dynamics Simulations

To simulate realistic experimental conditions, the MD simulations must be performed either at constant volume and temperature or at constant pressure and temperature. Unfortunately, the canonical and the isobaric-isothermal ensembles do not conserve the total energy. The conservation of a quantity, such as the volume or the pressure, requires a constant exchange of energy in between the macromolecular system and the surrounding heat bath. Such a process, in which the Liouville theorem is not valid, may be described in terms of non-Hamiltonian molecular dynamics [58], as will be detailed here. Firstly, we describe the general approach, which is subsequently applied to the canonical ensemble.

### 4.1. General Approach

To apply the abovementioned general method, we must complement the *N* Cartesian positions and momenta associated with the constituent atoms with *M* additional generalised coordinates and momenta. The set of all coordinates is collectively denoted by

(51) $$\begin{array}{c} \eta ={\left[r^{N}\;|\;p^{N}\;|\;q^{M}\;|\;p_{q}^{M}\right]}^{T}. \end{array}$$

It follows that the original Hamiltonian must be modified to include the generalised coordinates as well as an adjustable parameter *l*:

(52) $$\begin{array}{c} H\left(r^{N},p^{N}\right)⟶H^{\prime }\left(r^{N},p^{N},q^{M},p_{q}^{M};l\right). \end{array}$$

Unfortunately, the theory does not specify a particular form for the modified Hamiltonian. Because of the non-Hamiltonian nature of the dynamics, the Liouville theorem does not apply, which means that elementary volume elements are not conserved in phase space. The reason for this is that the modified dynamics have altered the geometry of the phase space from an Euclidean (flat) geometry to a Riemannian (curved) geometry. In Riemannian geometry, the Liouville theorem becomes [59]

(53) $$\begin{array}{c} \sqrt{\left|\det G\left(\eta \left(t\right)\right)\right|}d\eta \left(t\right)=\sqrt{\left|\det G\left(\eta \left(0\right)\right)\right|}d\eta \left(0\right), \end{array}$$

where **G**(**η**) is the metric associated with the Riemannian space, and det⁡ is the determinant of the matrix. We are only interested in the determinant of this matrix since only the latter is involved in the microcanonical partition function associated with the non-Hamiltonian system. The latter may be obtained directly from

(54) $$\begin{array}{c} \sqrt{\left|\det G\left(\eta \left(t\right)\right)\right|}=\exp\left[-\overset{t}{\underset{0}{\int }}\frac{\partial }{\partial \eta \left(\tau \right)}\cdot \dot{\eta }\left(\tau \right)d\tau \right]. \end{array}$$

The number of microstates associated with the non-Hamiltonian system may be written as

(55) ΩrN,pN,qM,pqM;l =Ω0∫dηdet⁡Gη∏k=1MδΛkη−Ck,

where $d\eta \sqrt{\left|\det G\left(\eta \right)\right|}$ is the invariant volume element in extended phase space and Λ_*k*_(**η**) − *C*_*k*_ is a function associated with a conservation law (for instance, the total energy, or the momentum associated with the barycentre of the system). If we integrate or marginalise the additional coordinates and momenta, we obtain a function that depends solely on the coordinates and momenta of the constituent atoms

(56) ΩrN,pN,qM,pqM;l→∫dqMdpqM∫dqMdpqMΩ′rN,pN;l.

The adjustable parameter is chosen in such a way that the marginalised microcanonical partition function is equal to the partition function of interest (for instance, the canonical (constant volume) partition function)

(57) ΩrN,pN,qM,pqM;l→∫dqMdpqM∫dqMdpqMΩ′rN,pN;l.

In the next section, we further clarify these notions by applying the general approach to the canonical ensemble.

### 4.2. Molecular Dynamics at NVT

In this section we outline the method to obtain the Hamiltonian that describes the canonical ensemble. This Hamiltonian may be substituted in the Liouville operator to obtain a finite difference equation. The modified Hamiltonian, called the Nosé-Hoover chain Hamiltonian [29, 60–62], is defined as

(58) $$\begin{array}{c} H_{\text{NHC}}=H\left(r^{N},p^{N}\right)+\overset{M}{\underset{i=1}{\sum }}\frac{p_{q_{i}}^{2}}{2\mu_{q_{i}}}+l\frac{q_{1}}{\beta }+\overset{M}{\underset{i=2}{\sum }}\frac{q_{i}}{\beta }, \end{array}$$

where

(59) $$\begin{array}{c} H\left(r^{N},p^{N}\right)=\overset{N-1}{\underset{i=1}{\sum }}\frac{p_{i}^{\prime 2}}{2m_{i}^{\prime }}+\frac{p_{⊙}^{2}}{2m_{⊙}}+U\left(r^{\prime N}\right) \end{array}$$

is the Hamiltonian of the macromolecule in barycentric coordinates, the primed variables are the barycentric coordinates (relative to the centre of mass), and ⊙ refers to the barycentre. If we assume that the energy of the modified Hamiltonian and the barycentre momentum are conserved (isolated system), we have, for the microcanonical partition function,

(60) ΩNHC =Ω0∫dpqMdqMdp′N−1dp⊙dr′N−1  ×exp⁡3N−2q1+∑i=2MqiδHNHC−C1  ×δeq1pΩ−C2.

If we marginalise the additional coordinate and momenta and choose *l* = 3*N*, the marginalised microcanonical partition function becomes equal to the canonical partition function, which means that the modified Hamiltonian describes the dynamics of the canonical ensemble

(61) $$\begin{array}{c} {\left.\Omega^{\prime }{\left(r^{N},p^{N}\right)}_{\text{NHC}}\right|}_{l=3N}\equiv Q\left(N,V,T\right). \end{array}$$

In the next section we focus on the constrained molecular dynamics.

### 4.3. Constrained Molecular Dynamics or How to Reduce the Computational Complexity

Macromolecules, such as the influenza hemagglutinin, possess many degrees of freedom. A typical influenza hemagglutinin is constituted of approximately 23.000 atoms, which means the macromolecules have typically 138.000 degrees of freedom in phase space (atomic positions and momenta). Unsurprisingly, the number of degrees of freedom increases dramatically if the structure is solvated. To reduce the computational complexity, it may be advantageous to impose constraints on certain degrees of freedom. Since the hydrogen atoms are light, they tend to follow the motion of heavier atoms quasi-instantaneously. Consequently, it is customary to fix their bonding length to reduce the computational burden. Let us assume that we have *K* holonomic (that depends only on time and coordinates) constraints:

(62) $$\begin{array}{c} \sigma_{k}\left(t\right)={\left\|r_{i}\left(t\right)-r_{j}\left(t\right)\right\|}^{2}-d_{k}^{2}\equiv 0. \end{array}$$

The constraints are enforced in the equations of motion with the method of Lagrange multipliers. Each constraint becomes a potential to which a variable is attached called a Lagrange multiplier *λ*_*k*_:

(63) $$\begin{array}{c} {\left.m_{i}{\ddot{r}}_{i}\left(t\right)=-\frac{\partial U\left(r^{N}\left(t\right)\right)}{\partial r_{i}}+\overset{K}{\underset{k=1}{\sum }}\lambda_{k}\frac{\partial \sigma_{k}}{\partial r_{i}}\right|}_{i=1,\ldots ,N}. \end{array}$$

Since the constraints are holonomic, they must be enforced at all times:

(64) $$\begin{array}{c} \sigma_{k}\left(t+\Delta t\right)\equiv 0. \end{array}$$

If we perform a truncated Taylor development of the previous equation, we may demonstrate that such a condition remains valid if a correction is applied to the Lagrange multiplier at each time step:

(65) $$\begin{array}{c} {\tilde{\lambda }}_{k}\left(t+\Delta t\right)={\tilde{\lambda }}_{k}\left(t\right)+\delta {\tilde{\lambda }}_{k}, \end{array}$$

where ${\tilde{\lambda }}_{k}\left(t\right)=\left(\Delta t^{2}/2\right)\lambda_{k}$ is the value of the Lagrange multiplier at time *t*, $\delta {\tilde{\lambda }}_{k}$ is the correction, ${\tilde{\lambda }}_{k}\left(t+\Delta t\right)$ is the value of the Lagrange multiplier at time *t* + Δ*t*, and

(66) $$\begin{array}{c} A\delta \tilde{\lambda }\approx -\sigma \left(r^{N}\left(t+\Delta t\right)\right), \end{array}$$

which are defined as

(67) $$\begin{array}{c} A=\left[A_{lk}\right]=\left[\overset{N}{\underset{i=1}{\sum }}\frac{1}{m_{i}}\frac{\partial \sigma_{l}\left(r^{N}\left(t+\Delta t\right)\right)}{\partial r_{i}}\cdot \frac{\partial \sigma_{k}\left(r^{N}\left(t\right)\right)}{\partial r_{i}}\right],\\ \delta \tilde{\lambda }=\left[\delta {\tilde{\lambda }}_{k}\right];\;\;\sigma =\left[\sigma_{k}\right]. \end{array}$$

In this the linear equation governing the corrections to the Lagrange multipliers in which **r** ^*N*^(*t*) are the* constrained* atomic coordinates at time *t*. Consequently, to obtain the correction, one must solve the linear equation associated with the correction.

Algorithms distinguish themselves by the approach they use to solve this equation. For the RATTLE algorithm, this equation is solved analytically [63]. For the SHAKE algorithm [64], only the diagonal elements of the **A** matrix are considered to reduce the complexity of the calculation, while for the MSHAKE algorithm [65] the whole matrix is solved with the LU decomposition. If the constraints are periodical, they must be expressed in terms of quaternions in which case the algorithm becomes QSHAKE [66].

In the next section, we consider a hybrid approach based on both molecular dynamics and Monte Carlo simulations.

### 4.4. Hybrid Monte Carlo Dynamics or How to Wisely Accelerate the Dynamics

In order to obtain realistic molecular dynamics simulations, the time increment must be kept small. Usually, the value of the time increment is chosen around 1 femtosecond (10^−15^ seconds), which is typically one to two orders of magnitude smaller than the time scale associated with the fastest molecular event, the bound-length vibration [48]. If one desires to explore slow molecular events, the time required to run the simulation may become rapidly prohibitive. For instance, the rotation of buried side chains takes about 10^−4^ to 1 second to complete, while an helix-coil transition may take as long as 10^4^ seconds. The situation might be even worse with macromolecular docking. Naturally, the time increment could be increased but the corresponding trajectory becomes rapidly unphysical.

This problem may be partially solved with an approach called hybrid Monte Carlo (HMC) simulation [28, 67]. As indicated by its name, HMC is a combination of MD and MC. The time evolution of the macromolecule is calculated with standard MD but with larger time increments. The outcome of a time iteration is accepted only if it is physically feasible. The feasibility of the outcome is assessed with a standard MC acceptance probability rule:

(68) ArNt,pNt⟶rNt+Δt,pNt+Δt=min⁡1,exp⁡−βHrNt+Δt,pNt+Δt−HrNt,pNtmmmmmmmiimm−HrNt,pNt.

Note that there is an importance subtlety associated with HMC. Because Newton's equations are time reversible (any trajectory may be reversed by inverting the direction of the motion), the transition probability must also be so [68]. That implies that the transition probability must be chosen so that

(69) TrNt,pNt⟶rNt+Δt,pNt+Δt =TrNt+Δt,−pNt+Δt⟶rNt,−pNt.

Otherwise, the HMC may generate unphysical results.

In the next section, we explore other ways that may be used to assess slow processes.

### 4.5. Computational Alchemy or How to Virtually Explore Experimentally Hidden Processes

Molecular dynamics is not limited to real physical potentials and equilibrium processes. Introducing an external, nonphysical, time-dependent, and possibly position-dependent potential may accelerate the dynamics of a slow process. This is called steered molecular dynamics or computational alchemy [69]. For instance, a force may be applied to a hemagglutinin to study potential conformational changes or to a drug (ligand) to analyse the docking process in between a drug and a neuraminidase. An example of how this could be achieved is by applying a random force (both in terms of amplitude and direction) to the barycentre of the ligand [70]. If the displacement of the ligand is above a certain threshold, the force is reapplied without modification. Otherwise, a new random force is generated. The whole process is repeated until the desired outcome is obtained. Such an approach is often combined with an immersive virtual environment to better visualise the outcome of the simulation [69]. In the next section, we explore an alternative to molecular dynamics and Monte Carlo simulations, namely, stochastic dynamics.

### 4.6. Molecular Dynamics and Influenza

There have been numerous applications of molecular dynamics in studies related to the influenza virus. For instance, molecular dynamics has been utilised for rational drug design for the influenza neuraminidase [7]. It has been instrumental to illustrate that the electrostatic funnel directs binding of Tamiflu to the influenza N1 neuraminidase [8]. Using molecular dynamics, it has been revealed that HR1039 is a potent inhibitor of the 2009 A (H1N1) influenza neuraminidase [9]. In addition, molecular dynamics has been valuable to show the role played by the BM2 channel in proton conductance and drug resistance for the influenza virus B [10]. Using molecular dynamics has made possible the design of inhibitor targeting drug-resistant mutants of the influenza A virus M2. Finally, conformational analysis of peptides and lipids of the influenza hemagglutinin fusion peptide in micelles and bilayer would not have been possible without the aid of molecular dynamics [14].

## 5. Langevin Dynamics, Self-Guide Langevin Dynamics, and Self-Guided Molecular Dynamics: Toward a Better Sampling of the Conformational Space

The dynamics of a macromolecular system is entirely determined by the potential *U*(**r** ^*N*^) associated with the process. For computational and practical reasons, this potential is virtually always an approximation of the real physical potential. Stochastic (random) dynamics attempts to bridge the gap in between the real and the approximate potential. Stochastic dynamics does not attempt to define the real potential but a general correction, which is independent on the particular details of the real potential. In other words, stochastic dynamics attempt to take into account the neglected degrees of freedom to obtain more realistic simulations, especially in the case of conformational sampling [71].

In this framework, it is assumed that each particle is under the influence of the potential *U*(**r** ^*N*^) and of a heat bath formed by the remaining *N* − 1 particles. Under general conditions using the Mori-Zwanzig theory [72] (one may obtain the same result by assuming that each particle is minimally coupled to a harmonic heat bath formed from the other particles), it is possible to demonstrate that the particles obey the generalised Langevin equation (GLE) [73]:

(70) mir¨it=−∂UrNt∂rimir¨it=−∫0tdτr˙iτγit−τ+ξiti=1,…,N,

where *γ*_*i*_(*t* − *τ*) is the memory kernel or dynamic friction and **ξ**_*i*_(*t*) is a stochastic term with mean:

(71) $$\begin{array}{c} \langle \xi_{i}\left(t\right)\rangle =0 \end{array}$$

and covariance:

(72) $$\begin{array}{c} \langle \xi_{i}\left(0\right)\xi_{j}\left(t\right)\rangle =2m_{i}k_{B}\gamma_{i}T\delta \left(t\right)\delta_{ij}I. \end{array}$$

Consequently, the correction is formed of two terms: a friction term, which introduces an artificial viscosity, and a stochastic term, which takes into account the unknown nature of the correction. One should notice that the frictional term depends on the previous history of the trajectory (Markovian process). The friction term is important in obtaining realistic simulations, as it takes into account the viscosity of the solvent (a feature, which is absent from both MD and MC). If one assumes that the frictional term is constant (no history), one obtains the celebrated Langevin equation (LE):

(73) $$\begin{array}{c} {\left.{\dot{p}}_{i}\left(t\right)=-\frac{\partial U\left(r^{N}\left(t\right)\right)}{\partial r_{i}}-\gamma_{i}{\dot{p}}_{i}\left(t\right)+\xi_{i}\left(t\right)\right|}_{i=1,\ldots ,N}. \end{array}$$

In that particular case, *γ*_*i*_ is simply called the frictional or damping term. The Langevin equation improves conformational sampling over standard molecular dynamics.

Conformational sampling may still be further improved if the trajectory history is reintroduced into the model [71]. To take history into account, a history-dependent guiding term Γ_*i*_(*t*) is added to the Langevin equation:

(74) $$\begin{array}{c} {\left.{\dot{p}}_{i}\left(t\right)=-\frac{\partial U\left(r^{N}\left(t\right)\right)}{\partial r_{i}}-\gamma_{i}{\dot{p}}_{i}\left(t\right)+\xi_{i}\left(t\right)+\lambda_{i}\Gamma_{i}\left(t\right)\right|}_{i=1,\ldots ,N}. \end{array}$$

This term could be defined in various manners. In self-guided Langevin dynamics (SGLD), the history-dependent guiding term is defined as the time average of the momentum over the last *l* iterations:

(75) ΓitSGLD =γipit¯t∈t−lΔt,t =1−ΔtlΔtΓit−Δt+ΔtlΔtγimir˙it−Δt2.

When, for self-guided molecular dynamics (SGMD), the history-dependent guiding term is defined as the time average of the potential plus its self-time average,

(76) ΓitSGMD =−∂UrNt∂ri+λiΓit−t∈t−lΔt,t =1−ΔtlΔtΓit−Δt  +ΔtlΔt−∂UrNt∂ri+λiΓit.

The guiding term is unphysical (as opposed to the memory kernel) and does not conserve energy. To reestablish energy conservation, an additional term *ς*(*t*)**p**_*i*_(*t*),   proportional to the momentum, must be added to the SGLD equation:

(77) p˙it=−∂UrNt∂ri−γip˙it +ξit+λiΓit−ςtpiti=1,…,N.

This term ensures the conservation of energy, providing that the running constant *ς*(*t*) obeys

(78) $$\begin{array}{c} \varsigma \left(t\right)=\frac{\sum_{i=1}^{N}\lambda_{i}\Gamma_{i}\left(t\right)\cdot {\dot{r}}_{i}\left(t\right)}{\sum_{i=1}^{N}p_{i}\left(t\right)\cdot {\dot{r}}_{i}\left(t\right)}. \end{array}$$

These equations may be easily integrated if they are recast under the form of Wiener processes [74]. The integration requires two independent Gaussian random variables.

Langevin dynamics has been used in many applications. For instance it has been instrumental to unravel the bilayer conformation of the fusion peptide of the influenza virus hemagglutinin [75]. Further, the use of Langevin dynamics aided researchers to characterise the loop dynamics and ligand recognition in human- and avian-type influenza neuraminidase [76].

In the next section, we address complexes stability such as an interaction between a drug and neuraminidase or that between monoclonal antibodies and hemagglutinin.

## 6. Free Energy, Binding, and Complexes or a Measure of Stability

The interaction in between a small molecule (drug) or an antibody with an antigen (hemagglutinin or neuraminidase) may result in a complex. It follows that, often, the stability of such a complex must be assessed. Indeed, if the complex is stable, the small molecule or the antibody may efficiently neutralise the corresponding antigen. Free energy [53] is a measure of such stability. Consequently, the calculation of free energy is addressed in the next section.

### 6.1. Thermodynamic Integration

The thermodynamics integration method [77] requires two states to determine the free energy: an unbound state *I* and a bound state *J*. From them, a parametric potential is defined as a linear superposition of the two potentials:

(79) $$\begin{array}{c} U\left(r^{N};\lambda \right)=\left(1-\lambda \right)U_{I}\left(r^{N}\right)+\lambda U_{J}\left(r^{N}\right). \end{array}$$

A corresponding parametric partition function is also defined as follows:

(80) $$\begin{array}{c} Z\left(N,V,T;\lambda \right)=\int dr^{N}\exp\left[-\beta U\left(r^{N};\lambda \right)\right]. \end{array}$$

Such a procedure is called adiabatic switching. The parametric derivative of the free energy may be obtained from the definition of the free energy:

(81) $$\begin{array}{c} \frac{\partial F\left(N,V,T;\lambda \right)}{\partial \lambda }=-\frac{1}{\beta }\frac{\partial }{\partial \lambda }\ln Z\left(N,V,T;\lambda \right). \end{array}$$

It follows that the difference of free energy in between the bound and the unbound state may be evaluated from

(82) $$\begin{array}{c} F_{J}-F_{I}={\overset{1}{\underset{0}{\int }}d\lambda \langle \frac{\partial U\left(r^{N};\lambda \right)}{\partial \lambda }\rangle }_{U\left(r^{N};\lambda \right)}. \end{array}$$

In the next section, we introduce a method based on irreversible work.

### 6.2. Nonequilibrium Method

In this section we describe an approach inspired from steered molecular dynamics (Section 4.5). As opposed to thermodynamic integration, the nonequilibrium method does not require the bound state. This state is reached as a result of the irreversible work effectuated by the synthetic time-dependent Hamiltonian. The nonequilibrium method is based on Jarzinski's equality and irreversible work [78]. Jarzinski's equality may be derived from a time-dependent Hamiltonian ensemble average and the Liouville theorem. From this equality, the difference in free energy in between a bound and an unbound state may be calculated from the ensemble average of the irreversible work applied to the system to bring the unbound state into a bound state:

(83) $$\begin{array}{c} F_{J}-F_{I}=-kT\ln{\langle \exp\left[-\beta W_{IJ}\left(r^{N},p^{N}\right)\right]\rangle }_{I}, \end{array}$$

where *W*_*IJ*_(**r** ^*N*^, **p** ^*N*^), the irreversible work, is given by

(84) $$\begin{array}{c} W_{IJ}\left(r^{N},p^{N}\right)=\overset{t_{J}}{\underset{t_{I}}{\int }}d\tau \frac{\partial H_{IJ}\left(r^{N},p^{N},t\right)}{\partial \tau }, \end{array}$$

and where the time-dependent Hamiltonian *H*_*IJ*_(**r** ^*N*^, **p** ^*N*^, *t*) is equal to the sum of the unbound macromolecular Hamiltonian *H*_*I*_(**r** ^*N*^, **p** ^*N*^) and of an external arbitrary time-dependent potential *U*_*IJ*_(**r** ^*N*^; *t*), which brings the macromolecular system from an unbound state *I* to a bound state *J*:

(85) $$\begin{array}{c} H_{IJ}\left(r^{N},p^{N},t\right)=H_{I}\left(r^{N},p^{N}\right)+U_{IJ}\left(r^{N};t\right). \end{array}$$

The ensemble average of the irreversible work is performed with the unbound Hamiltonian:

(86) exp⁡−βWIJrN,pNI =∫dpNdrNexp⁡−βWIJrN,pNmmimm×exp⁡−βHIrN,pN∫dpNdrNexp⁡−βWIJrN,pN  ×ZIN,V,T−1.

The next two sections present methods, which evaluate the free energy based on a subset of their generalised coordinates.

### 6.3. Blue Moon Ensemble Approach

Thermodynamic integration and the nonequilibrium method both exploit all the coordinates and momenta associated with the macromolecular system to evaluate the free energy. Correspondingly, one may evaluate the free energy from a subset called the reaction coordinates (e.g., bound length and bound angle) of the generalised coordinates such as the generalised coordinates directly associated with the binding process. This considerably reduces the complexity of the calculation but introduces a certain level of approximation, as most coordinates are neglected. In order not to clutter the notation, we will use only one reaction coordinate while keeping in mind that the generalisation of more than one coordinates is immediate.

The probability that a reaction coordinate *q*_*α*_ has a value *s* is

(87) $$\begin{array}{c} \mathrm{Pr}\left(s\right)=\frac{\int dp^{N}dr^{N}\exp\left[-\beta H\right]\delta \left(q_{\alpha }\left(r^{N}\right)-s\right)}{Z\left(N,V,T\right)}, \end{array}$$

where

(88) $$\begin{array}{c} q_{\alpha }=q_{\alpha }\left(r^{N}\right) \end{array}$$

is the representation of the reaction coordinates in terms of the Cartesian coordinates. The free energy associated with this coordinate is by definition

(89) $$\begin{array}{c} F\left(s\right)=-k_{B}T\text{lnPr}\left(s\right). \end{array}$$

If we marginalise (integrate) the momenta and if we express the potential in terms of the generalised coordinates, we obtain for the free energy [79]

(90) Fq=Fsi+∫siq∂UrNq3N∂qα−kBT∂ln⁡ det⁡ J∂qαqds,

where

(91) Oq=1ZN,V,Tδqα−s×∫dq3NOmm×exp⁡−βUrNq3NmmmmmmmrNq3N−kBTln⁡ det⁡ J−βUrNq3Nδqα−s,

and where the Jacobian matrix of the transformation is

(92) $$\begin{array}{c} J=\frac{\partial r^{N}}{\partial q^{3N}}. \end{array}$$

The main disadvantage of this approach is that all Cartesian coordinates must be converted into generalised coordinates, although only a subset of them (the reactions coordinates) are effectively exploited in the calculation of the free energy. This inefficiency is addressed in the next section.

### 6.4. Umbrella Sampling and Weighted Histogram Methods

When using umbrella sampling [80, 81] and the weighted histogram method [82] for the blue moon ensemble approach, the free energy is estimated from the reaction coordinates. The range (codomain and image) associated with the reaction coordinates is divided into a set of windows or intervals. A reaction coordinate distribution is associated with each window. The unbiased estimator for the probability of occurrence of a reaction coordinate* within a given window* is assumed to be the product of a biased Gaussian probability distribution and a biased correction based on an umbrella potential:

(93) Pr⁡q ≈12πσk2exp⁡−q−q−k22σk2exp⁡−βWq,sk,

where

(94) $$\begin{array}{c} W\left(q,s^{\left(k\right)}\right)=\frac{1}{2}\kappa {\left(q-s^{\left(k\right)}\right)}^{2} \end{array}$$

is the harmonic umbrella potential and *s* ^(*k*)^ is the centre of a given window, while ${\overset{-}{q}}_{k}$ and *σ*_*k*_^2^ are, respectively, the average and the variance of all the realisations of the reaction coordinates within window *k*. By definition, the derivative of the free energy associated with a particular window is equal to

(95) $$\begin{array}{c} \frac{dF_{k}}{dq}=-\frac{k_{B}T}{\mathrm{Pr}\left(q\right)}\frac{d\mathrm{Pr}\left(q\right)}{dq}=\frac{k_{B}T}{\sigma_{k}^{2}}\left(q-{\overset{-}{q}}_{k}\right)-\kappa \left(q-s^{\left(k\right)}\right). \end{array}$$

It is assumed that the derivative of the total free energy is a combination of the derivatives of the windows' free energy:

(96) $$\begin{array}{c} \frac{dF}{dq}=\overset{K}{\underset{k=1}{\sum }}C_{k}\left(q\right)\frac{dF_{k}}{dq}, \end{array}$$

and that the weighting coefficients are normalised

(97) $$\begin{array}{c} \overset{K}{\underset{k=1}{\sum }}C_{k}\left(q\right)=1. \end{array}$$

The full free energy profile may be extracted with numerical integration. In the next section, we describe another approach for the calculation of the free energy, which introduces a bias potential to better sample the reaction coordinates.

### 6.5. Metadynamics

As opposed to the previous methods, which were based on a single molecular dynamics simulation, the metadynamics approach involved two molecular dynamics simulations: the usual one in Cartesian coordinates under the influence of the real physical potential and a second one, the meta-simulation, which is performed under the influence of a biased potential. The bias potential is defined as

(98) UGrN=W∑t=τG,2τG,…exp⁡−∑α=1MqαrNt−qαrGNt22Δq2,

where *W* is a constant, *τ*_*G*_ is a time interval; **r** ^*N*^(*t*) is the time evolution (trajectory) of the complete set of Cartesian coordinates up to time *t* under the action of the real physical potential *U*(**r** ^*N*^), and **r**_*G*_^*N*^(*t*) is the trajectory of the system under the influence of both the physical potential and the biased potential *U*(**r** ^*N*^) + *U*_*G*_(**r** ^*N*^). From an analysis based on the Langevin equation, it is possible to demonstrate that the free energy may be obtained from

(99) $$\begin{array}{c} F\left(q^{M}\right)\approx W\underset{t=\tau_{G},2\tau_{G},\ldots }{\sum }\exp\left[-\overset{M}{\underset{\alpha =1}{\sum }}\frac{{\left(q_{\alpha }-q_{\alpha }\left(r_{G}^{N}\left(t\right)\right)\right)}^{2}}{2\Delta q^{2}}\right], \end{array}$$

in which the reaction coordinates are expressed in a function of the metacoordinates. The proof of this equation is beyond the scope of this review and may be found in [83]. As for umbrella sampling, only the reaction coordinates need to be expressed in terms of the Cartesian coordinates.

The use of free energy calculations has provided insights into the susceptibility of antiviral drugs against the E119G mutant of the 2009 influenza A (H1N1) neuraminidase [26]. It has also been utilized to show the impact of calcium on the N1 influenza neuraminidase [25].

In the next section, we explain how molecular dynamics may be combined with macromolecular docking to assert the dynamics of a macromolecular complex, such as a viral hemagglutinin, when interacting with a broadly neutralizing monoclonal antibody.

## 7. Binding and Docking or Computational Pharmacology and Vaccinology

From the outset, one may assume that the relative pose of the constituent of a complex may be obtained through molecular dynamics without resorting to macromolecular docking. Although this is, in principle, feasible, it is usually not realistic in practice. Indeed, the time scale associated with macromolecular docking is usually out of reach for most molecular dynamics simulations [48]. Larger time steps may be taken if a hybrid Monte Carlo approach is followed (Section 4.4), but most of the time, the increase is insufficient. For this reason macromolecular docking remains the favourite method to determine the relative pose in between a receptor and a ligand. (Note that macromolecular docking is beyond the scope of this paper, but a comprehensive algorithmic review may be found in [84]).

**Figure 2:**
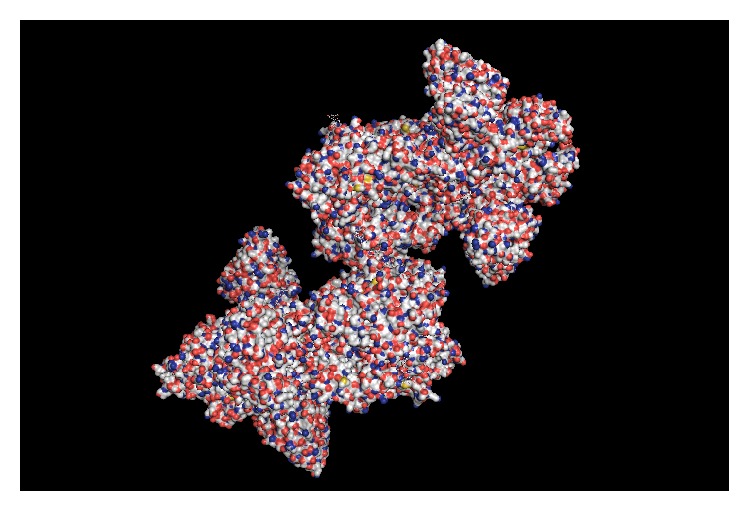
Molecular surface of influenza hemagglutinin (A/Viet Nam/1203/2004 (H5N1)) in complex with a broadly neutralizing antibody F10.

The abovementioned observation does not imply that MD is irrelevant for macromolecular docking. On the contrary, once the pose in between the receptor and the ligand has been determined through macromolecular docking, the dynamics of the resulting complex may be explored with MD simulations. Various dynamical aspects of the complex may be studied. These include the study of the conformational space associated with the receptor [85], binding path analysis [56], examining the structural changes associated with induced fit, studying the flexibility of the docking region, analysing transient binding, and aiding in structural refinement of the complex [86] as well as the design of drugs [87] and antibodies (see Figure 2) with optimal kinetic properties. MD may also be employed prior to macromolecular docking to generate conformations, which become the initial point for static docking. This is particularly beneficial if the ligand binds to conformations that rarely occur (relax complex method). Nevertheless, standard MD should not be utilised to explore low frequency motions associated with large conformational transformations. This is due to its limited ability to explore conformational change. Langevin dynamics may be more suited in that particular case. Also, from a computational point of view, multiple copies of the ligand may simultaneously interact with the receptor to search, in parallel, for the best docking region. There should be no interaction in between the copies. Molecular dynamics may also be utilised to evaluate the structural accessibility of a highly conserved epitope [4].

In the next section, we briefly explore the relationship in between quantum mechanics and molecular dynamics.

## 8. Path Integral or When Quantum Mechanics Meets Molecular Dynamics

The calculations that we have performed so far are based on classical and semiclassical approximations of quantum mechanics. Though being valid, these approximations are not as accurate as their quantum-mechanical counterpart [88]. However, quantum-mechanical (QM) methods are often computationally prohibitive. For that reason, quantum-mechanical methods are restricted to a small number of coordinates of interest (reaction coordinates) and the residual coordinates are analysed with molecular dynamics (the so-called hybrid quantum-mechanical molecular dynamics). Quantum mechanical methods are outside the scope of this review. More details may be found in [88]. Here, we limit ourselves to the connection in between quantum-mechanical methods and molecular dynamics. Quantum-mechanical methods are also based on the partition function formalism, which in the quantum case takes the form:

(100) $$\begin{array}{c} Q\left(N,V,T\right)=\mathrm{tr}\;\exp\left[-\beta \hat{H}\right], \end{array}$$

where tr⁡ is the trace operator and $\hat{H}$ is the quantum Hamiltonian operator, which may be obtained from the classical Hamiltonian as follows:

(101) $$\begin{array}{c} H\left(r_{i},\frac{\hbar }{i}\frac{\partial }{\partial r_{i}}\right)⟶\hat{H}. \end{array}$$

By extensively using the following identities:

(102) $$\begin{array}{c} \int dx|x\rangle \langle x|=\hat{I};\;\;\int dp|p\rangle \langle p|=\hat{I};\\ \langle x\;|\;p\rangle =\exp\left[\frac{\hbar }{i}px\right], \end{array}$$

and by making a certain number of approximations (Born-Oppenheimer approximation, Boltzmann statistics, and adiabatic approximation [83], etc.), it may be demonstrated that the partition function

(103) QN,V,T=∮Dr1τ⋯DrNτ ×exp⁡−1ℏ∫0βℏdτ12∑i=1Nmir˙i2τ+UrNτ

is equal to the path integral over all possible closed paths of the exponential of the classical Hamiltonian. The notation *D* **r**_*i*_(*τ*) means that we must integrate **r**_*i*_(*τ*) over all possible paths or trajectories. The resulting approach is known as path integral molecular dynamics [89]. Equation (103) involves only the atomics coordinates, but it is possible to introduce the conjugate momenta without altering the partition function by adding a harmonic potential to the Hamiltonian:

(104) QN,V,T =∮Dr1τ⋯DrNτDp1τDpNτ  ×exp⁡−1ℏ∫0βℏdτ∑i=1Npi22mi′τ  ×exp⁡−1ℏ∫0βℏ∑i=1N12miωK2ri2τ+UrNτ,

where

(105) $$\begin{array}{c} m_{i}^{\prime }=\frac{m_{i}K}{{\left(2\pi \hbar \right)}^{2}};\;\omega_{K}=\frac{\sqrt{K}}{\beta \hbar };\;K⟶\infty . \end{array}$$

The two equations are entirely equivalent. If we develop the notation, we obtain

(106) QN,V,T≈∫∏i=1Ndri1⋯driKdpi1⋯dpiK ×exp⁡−β∑k=1K∑i=1Npik22mi′+12miωK2rik+1−rik2mmmmm+1KUr1k⋯rNk∑k=1K∑i=1Npik22mi′+12miωK2rik+1−rik2riK+1=ri1∀i.

This path integral may be solved with the following infinite set of molecular dynamics equations:

(107) r˙ik=pikmi′k=1,…,∞,p˙ik=−miωK22rik−rik+1−rik−1p˙ik=−1K∂Ur1k⋯rNk∂rikk=1,…,∞.

Consequently, QM (at the approximation level indicated earlier) may be formulated in terms of an infinite (in practice, large) number of MD equations.

These equations may also be solved with an MC approach in which the position is sampled from a Gaussian distribution (the harmonic distribution in the path integral), and the new position is accepted or rejected according to a Metropolis rule based on the potential *U*. The sampling is made difficult by the fact that the positions are not statistically independent. To make them independent, the positions are expended in terms of their Fourier transform or their staging variables. When the staging variables are used, the partition function becomes

(108) QN,V,T≈∫∏i=1Ndui1⋯duiKdpi1⋯dpiK ×exp⁡−β∑k=1K∑i=1Npik22mi′k+12mikωK2uik2mimmnm+1KUr1ku1,…,rNkuN∑k=1K∑i=1Npik22mi′k+12mikωK2uik2uiK+1=ui1∀i,

where

(109) $$\begin{array}{c} r_{i}^{\left(1\right)}=u_{i}^{\left(1\right)},\\ r_{i}^{\left(k\right)}=u_{i}^{\left(1\right)}+\overset{K}{\underset{l=k}{\sum }}\frac{k-1}{l-1}u_{i}^{\left(k\right)},\;k=2,\ldots ,K,\\ m_{i}^{\left(1\right)}=0;\;m_{i}^{\left(k\right)}=\frac{k}{k-1}m_{i},\;k=2,\ldots ,K,\\ m_{i}^{\prime \left(1\right)}=m_{i};\;\;m_{i}^{\prime \left(k\right)}=m_{i}^{\left(k\right)}. \end{array}$$

As a result, each normal mode **u**_*i*_^(*k*)^ may be sampled from an independent Gaussian distribution and the positions may be reconstructed from their corresponding staging transformations. For instance, path integrals have been instrumental to underline the importance of quantum effects in the influenza neuraminidase [90].

## 9. Conclusions

The conformational space and the dynamics associated with macromolecules and macromolecular complexes, as well as their structural stability, may be explored and asserted through molecular dynamics and Monte Carlo simulations. Although full knowledge of the algorithms involved is not absolutely required, to obtain meaningful simulations, it is impressible to understand the conditions, approximations, and hypotheses on which they are based, as well as their limitations and potentialities.

Although most simulations are currently limited to individual structures or small organisms and complexes (a few millions atoms), it is conceivable that in the near future large viruses, such as the influenza, may be entirely simulated in silico over long periods of time. That would open the door to an essentially in silico design and lead to a better assessment of toxicity, specificity, and efficiency. Such a phenomenon has occurred before in aerodynamics in which computational fluid dynamics has essentially replaced wind tunnel experiments in the design of large aeroplane fuselages and wings.
